# Subunit vaccine candidates against *Aeromonas salmonicida* in rainbow trout *Oncorhynchus mykiss*

**DOI:** 10.1371/journal.pone.0171944

**Published:** 2017-02-09

**Authors:** Moonika Haahr Marana, Louise von Gersdorff Jørgensen, Jakob Skov, Jiwan Kumar Chettri, Andreas Holm Mattsson, Inger Dalsgaard, Per Walter Kania, Kurt Buchmann

**Affiliations:** 1 Department of Veterinary Disease Biology, Faculty of Health and Medical Sciences, University of Copenhagen, Denmark; 2 National Veterinary Institute, Technical University of Denmark, Denmark; 3 Evaxion Biotech, Copenhagen, Denmark; Universita degli Studi della Tuscia, ITALY

## Abstract

*Aeromonas salmonicida* subsp. *salmonicida* is the etiological agent of furunculosis and a major fish health problem in salmonid aquaculture worldwide. Injection vaccination with commercial mineral oil-adjuvanted bacterin vaccines has been partly successful in preventing the disease but in Danish rainbow trout (*Oncorhynchus mykiss*, Walbaum) aquaculture furunculosis outbreaks still occur. In this study we tested the efficacy of experimental subunit vaccines against *A*. *salmonicida* infection in rainbow trout. We utilized *in silico* screening of the proteome of *A*. *salmonicida* subsp. *salmonicida* strain A449 and identified potential protective protein antigens that were tested by *in vivo* challenge trial. A total of 14 proteins were recombinantly expressed in *Escherichia coli* and prepared in 3 different subunit vaccine combinations to immunize 3 groups of rainbow trout by intraperitoneal (i.p.) injection. The fish were exposed to virulent *A*. *salmonicida* 7 weeks after immunization. To assess the efficacy of the subunit vaccines we evaluated the immune response in fish after immunization and challenge infection by measuring the antibody levels and monitoring the survival of fish in different groups. The survival of fish at 3 weeks after challenge infection showed that all 3 groups of fish immunized with 3 different protein combinations exhibited significantly lower mortalities (17–30%) compared to the control groups (48% and 56%). The ELISA results revealed significantly elevated antibody levels in fish against several protein antigens, which in some cases were positively correlated to the survival.

## Introduction

*Aeromonas salmonicida* subsp. *salmonicida* (hereafter AS) is the causative agent of typical furunculosis in aquacultured salmonid fish. Furunculosis causes bacterial septicemia that leads to significant economic losses due to fish morbidity and mortality [1].

Vaccination of salmonids against furunculosis is generally applied with injection vaccines containing formalin-killed AS bacteria combined with mineral oil adjuvant. These vaccines provide efficient protection and induce long-lasting immunity against the bacterium under certain conditions [2]. However the prophylactic effect of the vaccines in Danish rainbow trout is suboptimal under field conditions and the maricultured fish still experience furunculosis outbreaks during the warmer summer months [3, 4]. Furthermore, adverse side-effects like intra-abdominal lesions, retarded growth, pigmentation and autoimmunity [2, 5–9] have been associated with vaccine administration. Consequently, research for developing more effective furunculosis vaccines with fewer side-effects is ongoing.

The bacterium of AS, first described in 1894 [10] is one of the most important and extensively studied fish pathogens. Important virulence factors identified in AS comprise the A-layer protein VapA [11–14], several iron-regulated outer membrane proteins (IROMPs) [15–19], extracellular protein complexes including serine protease AspA and lipase CGAT with LPS [20–23] and the type three secretion system T3SS [24] consisting of effector and structural proteins essential for AS virulence [25–27]. The potential of these pathogenic and virulence factors as vaccine candidates has been investigated in challenge trials previously [15, 28–36].

In this study we applied an *in silico* approach to select potential vaccine candidates for experimental furunculosis vaccines and selected 14 proteins for *in vivo* trial. The proteins were recombinantly expressed in *E*. *coli* and prepared in 3 different vaccine combinations to immunize groups of rainbow trout by intraperitoneal (i.p.) injection. The vaccine efficacy was assessed by infection trial and by measuring the antibody reactivity in immunized fish on grounds that the antibody response has in several studies confirmed to be closely correlated to protection [14, 37, 38].

## Materials and methods

### Rationale selection

The functionality and domain classification of 14 proteins (Table 1) was conducted by InterPro [39]. The subcellular localization of the individual proteins was predicted by the CELLO and pSORTb predictor [40, 41] that provide an output with reliability score for each location of each protein. Commonly, protective B-cell protein antigens are located in the outer membrane and extracellular environment, hence these predicted subcellular locations were targets for selection [42, 43]. The conservation study was done by local sequence alignment (Smith–Waterman algorithm) [44] based on the amino-acid sequence of the protein antigens tested for conservation across the public available NCBI chromosome data. The pair-wise alignment values (% similarity and % coverage) were calculated and the most similar protein to a given genome was selected and classified as homologous if the % similarity was >75% on at least 75% of the total protein length (% coverage). Protein conservation correlates with an increased probability of success, due to the ability to elicit protection across different bacterial strains [45]. Therefore, we performed a conservation analysis of the initially selected *A*. *salmonicida* proteins across the identified incomplete chromosome genome sequences available in the NCBI database. The primary strain (complete chromosome from NCBI) was strain A449 [46]. The 4 sub-strains (incomplete chromosomes from NCBI) were strain: 01-B526, CBA100, NBRC_13784 and pectinolytica_34mel.

**Table 1:** Rationale for protein selection.

PROTEIN ID	RATIONALE OF SELECTION			FUNCTION	LOCATION		CONSERVATION (NCBI’s incomplete chromosomes)			
	F	L	C	InterPro server	PSORTb server	CELLO server	% lc	h/s	M % sim.	SD %
ASA_3320	x		x	extracellular enzyme activity	unknown	periplasmic	100	[4/4]	98	1
ASA_0826	x	x		virulent enzymatic activity	extracellular	extracellular	50	[2/4]	99	1
ASA_3455	x	x	x	extracellular enzyme activity	extracellular	extracellular	50	[2/4]	99	1
ASA_3883	x	x	x	iron sideophore receptor	outer membrane	outer membrane	100	[4/4]	98	2
ASA_2321	x		x	heme binding	periplasmic	periplasmic	100	[4/4]	98	2
ASA_0744	x		x	pathogenesis, membrane	cytoplasmic	periplasmic + cytoplasmic	100	[4/4]	97	2
ASA_1342	x	x		motility	extracellular	periplasmic	75	[3/4]	97	3
ASA_2532	x		x	motility	unknown	periplasmic	100	[4/4]	96	5
ASA_4042			x	-	periplasmic	periplasmic	100	[4/4]	92	9
ASA_P5G035	x	x		adhesion, pilin assembly	outer membrane	extracellular + outer membrane	50	[2/4]	97	1
ASA_1675	x	x	x	virulent enzymatic activity	extracellular	extracellular	50	[2/4]	98	1
ASA_3328	x	x	x	haemoglobin/transferrin/lactoferrin receptor	outer membrane	outer membrane	100	[4/4]	98	3
ASA_3723	x	x	x	collagenase	extracellular	outer membrane + cytoplasmic	100	[4/4]	99	2
ASA_4105		x	x	zinc metallopeptidase	outer membrane	outer membrane	100	[4/4]	98	2

Fourteen proteins of *A*. *salmonicida* subsp. *salmonicida* were selected as potential protective B-cell antigens. The column RATIONALE OF SELECTION shows which criteria were responsible for the selection: functionality (F), location (L), conservation (C). E.g. protein ASA_2532 was selected due to its predicted motility function domain by InterPro and its strong conservation. Besides being present in the primary strain A449 (complete chromosome), it was represented in all NCBI’s incomplete chromosomes: number of homologs/number of strains (h/s). The % library coverage (% lc) of protein ASA_2532 is 100% meaning that this protein is 100% conserved across all sub-strains. Mean of % similarities to homologs (M % sim.) is 96% meaning this protein has an average % identity of 96% to the homologs of the 4 sub-strains. SD is the standard deviation of % similarities to homologs.

### Recombinant construct design

The 14 proteins were expressed in constructs based on conventional *in silico* analysis such as prediction of signal peptides (SignalP-4.1) [47], transmembrane regions (TmHmm-2.0) [48], non-classical secretion proteins (SecretomeP-2.) [49], functional and structural domains (InterPro [39] and DomCut [50]) (Table 2). The rationale of expressing the protein in fragments was due to: 1) enhanced probability of expressing the native protein structure, 2) expressing the protective part of the protein, 3) establishing a successful recombinant expression in *E*. *coli*. Predicted signal peptide or/and transmembrane and intracellular regions were removed. Proteins bigger than 1000aa were split in N- and C-terminal fragments and only the fragment comprising the predicted active functional site domains was expressed. The splitting point of these fragments where decided based on structural domain predictor aiming at keeping the native structure of each structural domain.

**Table 2:** Recombinant construct design, protein type and vaccine formulation.

Locus ID	Expressed aa-region	Predicted protein type	Vaccine group	Reference	Removed sequences	
					SP	TM & IC
ASA_3320	24–640	endochitinase (chiB)	VacA	[46, 62,64]	x	
ASA_0826	2340–3195	RTX protein (asx)(only active site)	VacA	[46, 62,64]		
ASA_3455	25–754	alpha-amylase (amyA)	VacA	[46]	x	
ASA_3883	25–680	outer membrane ferric siderophore receptor	VacA		x	
ASA_2321	22–480	cytochrome c-552 (nrfA	VacA		x	
ASA_0744	30–388	tolA protein (tolA)	VacB			x
ASA_1342	1–639	polar flagellar hook-length control protein (FliK)	VacB	[46]		
ASA_2532	23–334	type IV pilus assembly protein TapV (tapV)	VacB	[65,66]	x	x
ASA_4042	19–402	hypothetical uncharacterized protein	VacB		x	
ASA_P5G035	24–629	conjugal transfer mating pair stabilization protein (TraN)	VacB		x	x
ASA_1675	310–1156	hemolysin-type calcium-binding protein (only active site)	VacC	[62]		
ASA_3328	25–697	putative heme receptor (hupA)	VacC	[16, 46, 63]	x	
ASA_3723	210–915	microbial collagenase (only active site)	VacC	[46,62, 64]	x	
ASA_4105	35–468	M24/M37 family peptidase	VacC		x	

All proteins are listed by their protein ID, expressed amino acid sequence (aa-region), predicted function, vaccine group, reference and construct design showing if the sequence removed was a signal peptide (SP) or transmembrane (TM) and intracellular (IC) region.

### Protein expression

All recombinant protein constructs were expressed in *E*. *coli* by Creative Biomart (Shirley, NY, USA).

### Vaccine preparation

The 14 recombinant protein constructs were allocated in 3 groups as follows: VacA and VacB (both 5 proteins) and vacC (4 proteins) (Table 2). A total of 25 μg of an individual protein was prepared per fish in the vaccine mixture corresponding to a total of 100–125 μg of mixed proteins per fish. The proteins were allowed to bind to Al(OH)_3_ by adding aluminium hydroxide gel adjuvant Alhydrogel (Brenntag, Denmark) to each vial of mixed proteins. A volume of 100 μL Al(OH)_3_ was added per 160 μg protein and incubated at room temperature with end-over-end rotation for 1 hour. The vials were then centrifuged at 216 *G* (Heraeus multifuge 3 L-R, Thermo Scientific, Denmark) at room temperature for 2 minutes. The supernatant was removed and checked for the absence of proteins with NanoDrop 2000 (Thermo Scientific, Denmark). The vials containing proteins bound to Al(OH)_3_ were washed twice with 0.9% sterile NaCl—each time all vials were centrifuged at 216 *G* for 2 minutes at room temperature whereafter the supernatant was removed. After the last wash an equal volume of Freund’s Incomplete Adjuvant (FIA) (F5506, Sigma-Aldrich, Denmark) was added and the vials were vigorously vortexed at room temperature for 1 hour. The vaccine control containing only adjuvants was made accordingly without the protein constructs. The vaccines were stored at 4°C until the further use.

### Fish

Disinfected eyed rainbow trout eggs originating from Fousing Trout Farm, Jutland, Denmark were translocated and hatched in a pathogen-free rearing facility at Bornholm Salmon Hatchery (AquaBaltic, Denmark). Details of retaining the pathogen-free status have previously been described [51]. Fish were immunized and then reared for 6 weeks (568 degree-days) in the system containing recirculated municipal water at 13–14°C in 700 L (1 m^3^) tanks and fed 1% biomass per day with dry pellet feed (BioMar A/S, Denmark). Fish were transported to the fish keeping facility at the University of Copenhagen, Frederiksberg, Denmark and acclimatized by gradually raising the water temperature to 19°C over the course of the week before the challenge (exposure to live AS) was performed. The study was approved under the license no. 2015-15-0201-00655 issued by the Animal Experiments Inspectorate, Ministry of Environment and Food, Denmark.

### Vaccination

A total of 360 rainbow trout (average body weight of 30 g) were randomly divided into 6 groups of 60 fish (Table 3) and each group was further subdivided into duplicate tanks containing 30 fish each. The fish were anaesthetized (75 mg L^-1^ MS222, Sigma-Aldrich, Denmark) and i.p. injected with 0.1 mL of different formulations warmed up to fish body temperature (14°C) prior the immunization. The different vaccine formulations included: 1) commercial furunculosis vaccine Alpha Ject^®^ 3000 (Pharmaq AS, Norway), 2) VacA, 3) VacB, 4) VacC (the experimental subunit vaccines consisting of *in silico* predicted proteins), 5) adjuvant only (the equal amount of Alhydrogel and FIA) and 6) saline.

**Table 3:** Experimental setup presenting the different groups, sample size, mortality and relative percentage of survival (RPS).

	Control Saline	Control Adjuvant	Alpha Ject® 3000	VacA	VacB	VacC
No. of immunized fish	60	60	60	60	60	60
No. of fish sampled for ELISA (7wpi)	10	10	10	10	10	10
No. of challenged fish	50	50	50	50	49*	48*
Mortality %	56	48	8	30	20	17
RPS	-	14	86	46	64	70
No. of fish sampled for ELISA (3wpc)	10	10	10	10	10	10

* Fish, one and two, respectively, died after anaesthesia during the challenge infection.

### Challenge experiment

At 7 weeks post-immunization (wpi) (690 degree-days) the 25 fish (now with an average body weight of 40 g) in all the duplicate tanks were challenged with 5.4×10^8^ CFU mL^-1^ AS strain 090710-1/23 by a challenge method using a multi-puncture device [37] producing 10 perforations in the tail fin with subsequent exposure to bacteria, for 90 seconds. Morbidity was monitored every second hour during 3 weeks post-challenge (wpc). The moribund state was defined as a complete loss of equilibrium, strong discoloration and development of skin hemorrhages. Moribund fish were immediately removed for euthanasia (300 mg L^-1^ MS222) and recorded as mortalities. Swabs from head kidney of all freshly euthanized fish were plated onto 5% blood agar plates (SSI Diagnostica, Denmark) for bacteriological analysis. The confirmation of AS was done according to Dalsgaard and Madsen [4] and the re-isolated bacteria from the dead fish were confirmed as *A*. *salmonicida* subsp. *salmonicida*. The challenge trial was terminated 3 wpc (24 days).

### Sampling

Blood samples for ELISA (10 fish per group) were taken after vaccination (6 wpi) and after challenge (3 wpc) (Table 3). Blood were collected by caudal vein puncture from euthanized fish (300 mg L^-1^ MS222), allowed to clot at 4°C overnight and serum was separated at 4°C by centrifugation (3000 *G*) for 10 minutes and stored at -80° C until further analysis.

### ELISA

Enzyme-linked immunosorbent assay (ELISA) was performed according to previously established protocol [52]. In brief, the 96-well microtiter plates (MaxiSorp™, Nunc, Denmark) were coated overnight at 4°C with either sonicated lysate of AS bacteria strain 090710-1/23 (protein conc 5 μg mL^-1^) or individual recombinant proteins (conc 1 μg mL^-1^) and blocked with 2% Bovine Serum Albumin (BSA, A4503, Sigma-Aldrich, Denmark) for 1 hour at room temperature. A total of 9 proteins (contained in VacB and VacC vaccines) showing best protection were chosen for plate coating for ELISA. Working dilutions were selected considering preliminary results. Dilutions 1:500 and 1:5000 were chosen for testing specific antibodies against sonicated bacteria and dilutions 1:100, 1:1000 and 1:10 000 were chosen for testing specific antibodies against individual proteins. All serum samples were diluted with assay diluent (0.1% BSA in wash buffer) and 100 μL of sample was added to each duplicate ELISA plate well and incubated at 4°C overnight. The plates were thereafter incubated with 100 μL mouse anti-salmonid Ig (MCA2182 AbD Serotec, Germany, diluted 1:500) and 100 μL HRP-conjugated rabbit anti-mouse IgG for 1 hour (STAR13B, AbD Serotec, Germany, diluted 1:500). The color reaction was developed with 100 μL tetramethylbenzidine (TMB) PLUS substrate (BUF042A, AbD Serotec, Germany) and stopped after 10 minutes by adding 100 μL 1N HCL. All ELISA plates included 4 wells of pure assay diluent for the background absorbance measurement. The optical density (OD) was measured at 450 nm in an Epoch spectrophotometer (BioTek, USA) in duplicate wells.

### Statistical analysis

The relative percentage of survival (RPS) was calculated as proposed by Amend [53]: RPS = [1-(% mortality in vaccinated fish / % mortality in control fish)] × 100. All statistical tests were performed using GraphPad Prism version 4.00 for Windows (GraphPad Software, USA, www.graphpad.com) and P-values < 0.05 were considered statistically significant. Mortality data was analyzed using Kaplan-Meier survival analysis and log-rank test. Duplicate groups were pooled after survival curve comparison showed no significant difference in mortalities. ELISA results were compared using one-way ANOVA followed by Tukey’s Multiple Comparison post hoc test. Fish from duplicate tanks were pooled after t-test had confirmed no significant difference between them. Correlation was calculated as Pearson correlation coefficient (r).

## Results

### Challenge experiment

**Fig 1:**
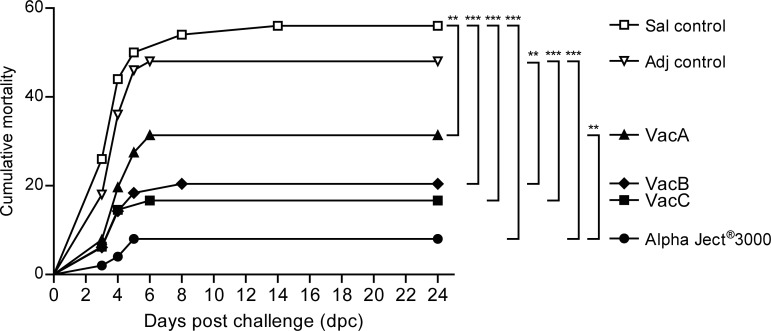
The cumulative mortality in rainbow trout experimental groups (replicates pooled) following infection challenge with *A*. *salmonicida*. Asterisks (*) represent p values between groups (*p<0.05, **p< 0.01, ***p< 0.001).

All fish were challenged at 7 wpi by multi-puncture of the upper caudal fin and local exposure to a known number of AS. The mortality post-challenge (pc) is shown in Fig 1. Mortality started at day 3 pc in all fish groups and subsided by day 6 pc. Thereafter minor mortality still occurred until day 15 pc. The highest cumulative mortality was recorded in the saline control group (56%) followed by the adjuvant only control group (48%). Mortalities in the experimental subunit vaccine groups VacA (30%, RPS 46), VacB (20%, RPS 64) and VacC (17%, RPS 70) were significantly lower than in the saline control group. The lowest cumulative mortality, but not significantly different form VacB and VacC groups, occurred in the group immunized with the commercial vaccine Alpha Ject^®^ 3000 (8%, RPS 86).

### ELISA

ELISA analyses were conducted to measure the antibody response towards sonicated bacteria and individual recombinant proteins in different groups of immunized fish. Here we highlight only significant differences (p<0.05) in antibody response that occurred when comparing the immunized fish groups (Alpha Ject^®^ 3000, VacA, VacB, VacC) with the control fish groups (saline control and adjuvant control).

#### Antibodies against sonicated AS

**Fig 2:**
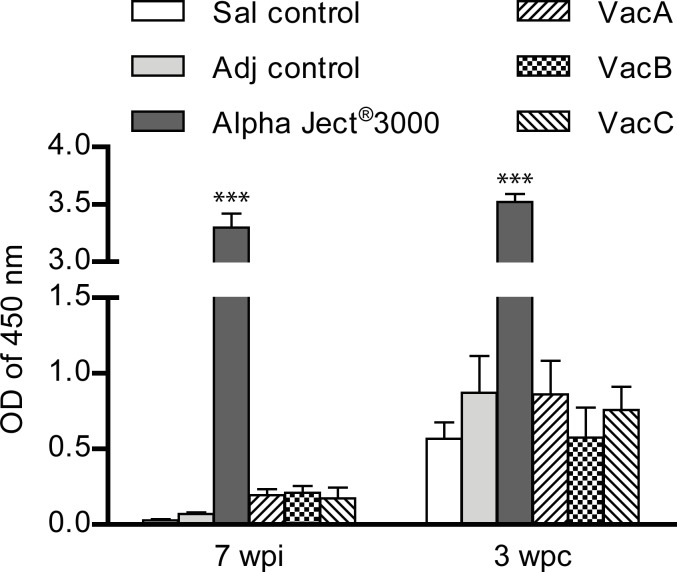
Levels of *A*. *salmonicida*-specific antibodies in serum measured by ELISA at 7 weeks post-immunization (wpi) and 3 weeks post- challenge (wpc). Sera (diluted 1:500) were analyzed from 10 fish per group (5 fish per duplicate tank). Asterisks (*) represent p values (*p<0.05, **p< 0.01, ***p< 0.001) compared to saline control group.

Antibody levels against sonicated bacteria in sera (diluted 1:500) from fish vaccinated with Alpha Ject^®^ 3000 were significantly higher compared to all other groups at 7 wpi and 3 wpc (Fig 2). A slight but non-significant antibody increase against sonicated bacteria was seen in the groups immunized with the subunit vaccines when compared to the control groups.

#### Antibodies against 9 recombinant proteins

**Fig 3:**
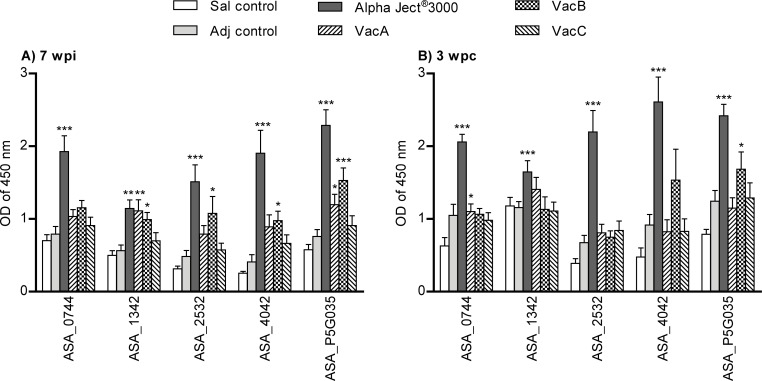
Levels of antibodies in serum of fish against individual VacB vaccine proteins. Sera (diluted 1:100) was measured by ELISA from 10 fish per group (5 fish per duplicate tank) at 7 weeks post-immunization (wpi) (a) and at 3 weeks post-challenge (wpc) (b). Asterisks (*) represent p values (*p<0.05, **p< 0.01, ***p< 0.001) when compared to saline control group.

**Fig 4:**
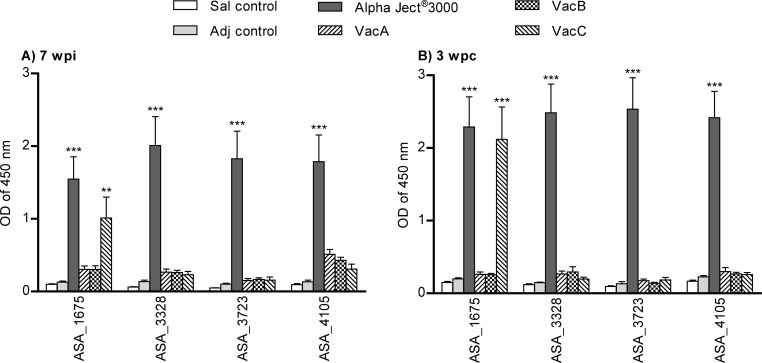
Levels of antibodies in serum of fish against individual VacC vaccine proteins. Sera (diluted 1:100) was measured by ELISA from 10 fish per group (5 fish per duplicate tank) at 7 weeks post-immunization (wpi) (a) and at 3 weeks post-challenge (wpc) (b). Asterisks (*) represent p values (* = P<0.05, ** = P< 0.01, *** = P< 0.001) when compared to saline control group.

The fish immunized with the Alpha Ject^®^ 3000 had significantly higher antibody response against all 9 recombinant proteins when compared to the control groups at 7 wpi and 3 wpc. Fish immunized with experimental vaccines (recombinant proteins) showed diverse antibody responses to these proteins (Figs 3 and 4).

#### Antibodies against proteins in VacB vaccine

Significantly higher antibody levels were found in fish immunized with experimental vaccine VacB containing recombinant proteins ASA_0744, ASA_1342, ASA_2532, ASA_4042, ASA_P5G035 compared to the saline injected control group. This group showed significantly higher serum antibody levels (diluted 1:100) against proteins ASA_1342, ASA_2532, ASA_4042, ASA_ P5G035 at 7 wpi (Fig 3A) and against ASA_P5G035 at 3 wpc (Fig 3B). In addition, fish immunized with experimental vaccine VacA (ASA_3320, ASA_0826, ASA_3455, ASA_3883, ASA_2321) had significantly higher antibody levels in sera (1:100) against few vaccine VacB proteins: ASA_1342 and ASA_P5G035 after immunization (7 wpi) (Fig 3A) and ASA_0744 after challenge (3 wpc) (Fig 3B).

#### Antibodies against proteins in VacC vaccine

Fish immunized with peptides of experimental vaccine VacC (ASA_1675, ASA_3328, ASA_3723, ASA_4105) had significantly elevated antibody levels in sera (diluted 1:100) against ASA_1675 at both 7 wpi (Fig 4A) and 3 wpc (Fig 4B) when compared to the control groups.

### Correlation between antibody levels and survival

**Fig 5:**
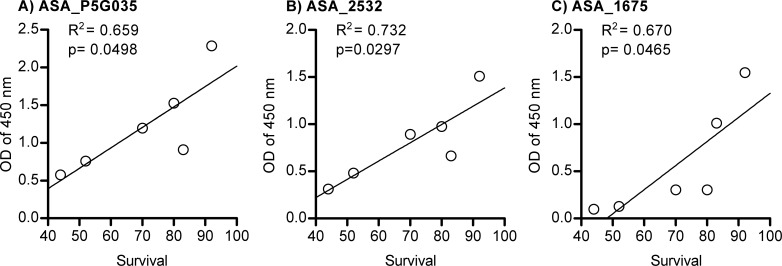
Correlation between mean antibody (diluted 1:100) levels for individual proteins at 7 weeks post-immunization (wpi) and survival in different experimental groups at 24 days post-challenge (dpc). Antibody levels were measured from 10 fish per group (5 fish per duplicate tank) by ELISA one day before challenge.

The correlation between serum antibody (diluted 1:100) levels against recombinant proteins at 7 wpi and the survival at day 24 pc was calculated as Pearson correlation coefficient (r). A significant (p<0.05) positive correlation was found for antibody reactivity against VacB proteins ASA_2532 (Pearson r = 0.86; R^2^ = 0.73), ASA_P5G035 (Pearson r = 0.81; R^2^ = 0.66) and VacC protein ASA_1675 (Pearson r = 0.81; R^2^ = 0.67) (Fig 5).

## Discussion

This study utilized recombinant proteins encoded by the genome of *A*. *salmonicida* subsp. *salmonicida* A449 for immunization against furunculosis. The *in silico* approach also known as reverse vaccinology [54] has been used to identify vaccine candidates for bacterial fish diseases in few studies previously [55–57] but to the best of our knowledge—this is the first time it has been applied for *A*. *salmonicida* subsp. *salmonicida*. The 14 antigens selected for this study were presumed to be associated with hemoglobin binding, adhesion, iron-acquisition, calcium and siderophore binding, motility, enzymatic and collagenase activity and possess the right characteristics to be surface exposed proteins. The protective properties of these 14 recombinant protein antigens were tested by *in vivo* challenge experiment. The experimental setup included 3 groups of fish immunized with protein antigens, one group immunized with a commercial vaccine Alpha Ject^®^ 3000 and 2 control groups injected with either saline or adjuvant only. Antigens administered in experimental subunit vaccines induced protection in the immunized fish. Thus, all the groups of fish immunized with experimental vaccines experienced significantly lower mortalities compared to the saline control group or the group injected with adjuvant (Fig 1). The fish vaccinated with Alpha Ject^®^ 3000 had significantly higher antibody levels against sonicated bacteria when compared to all the other groups (Fig 2) and performed best (mortality 8%) throughout the challenge study but not significantly better compared to the vaccine groups VacB and VacC (20% and 17% mortality, respectively). The experimental vaccine group VacA exhibited the highest mortality (30%) among the groups immunized with *in silico* predicted antigens but the mortality was still significantly lower compared to the control fish injected with saline (56%).

The ELISA analysis measuring antibody response against the individual proteins showed that fish vaccinated with the commercial vaccine had significantly higher antibody levels against all individual proteins included in experimental vaccines VacB and VacC when compared to the saline control group. The fish immunized with the experimental vaccine VacC reacted with elevated antibody levels against ASA_1675—a protein that was included in the VacC vaccine formulation. Antibody reactivity against ASA_1675 was significantly higher both after the immunization and challenge when compared to the control groups and other experimental vaccine groups (Fig 4). This suggests that ASA_1675 is highly immunogenic and may play a role in protection considering VacC vaccine fish exhibited the lowest mortality among the experimental vaccine groups and that the antibody levels against ASA-1675 measured before challenge were positively correlated with the fish survival (Fig 5). However as the experimental vaccines consisted of different proteins, their individual contribution to vaccine induced protection is uncertain and the role of ASA_1675 should be assessed in further studies. The protein ASA_1675 is a hemolysin-type calcium binding repeat-containing protein belonging to a family of putative RTX (repeats-in-toxin) proteins involved in various biological functions like S-layer formation, adhesion, pore formation, hemolysis, cytolysis etc. [58]. The RTX family proteins are secreted by the type I secretion system (TISS) of Gram negative bacteria and have shown to be important virulence factors in various bacteria [58, 59] including *Aeromonas hydrophila* ATCC 7966^T^ [60] and *Vibrio anguillarum* [61]. The secretion of the protein in AS seems to occur only *in vivo* [62]. Other proteins in VacC vaccine formation mentioned previously in relation to AS virulence [46] are ASA_3328 and ASA_3723. The ASA_3328 is a putative heme receptor (hupA) involved in heme utilization [63] and has observed exclusively up-regulated under *in vivo* and in iron-restricted conditions *in vitro* [16]. The ASA_3723 is a microbial collagenase orthologue to a putative virulence determinant AHA_0517 in *A*. *hydrophila* [60] and among one of the most secreted proteins in the supernatant of virulent AS [62, 64]. The ASA_4105 is a M24/M37 family zinc metallopeptidase and has not been previously mentioned as potential virulent factor of AS but was chosen in VacB vaccine due to its outer membrane location and the conservation across *A*. *salmonicida* sub-strains.

The fish that were immunized with the VacB vaccine exhibited significantly higher antibody levels against 4 out of 5 proteins they were immunized with (Fig 4) but the antibody reactivity against these proteins was not as distinguished as the antibody response against ASA_1675 in fish vaccinated with VacC vaccine. The VacB proteins inducing higher antibody response in fish vaccinated with VacB vaccine included ASA_1342, ASA_2532, ASA_4042 and ASA_P5G035. ASA_1342 is a the polar flagella hook-length control protein (FliK) that has previously been mentioned as potential virulent factor of AS having homologues to flagella proteins in *A*. *hydrophila* [46]. ASA_2532 is a type IV pilus assembly protein (TapV) shown to contribute to virulence of AS by being engaged in bacterial adhesion [65] and is homologues to FimV in *Pseudomonas aeruginosa* [66] and similar to pilus assembly proteins in other pathogenic bacteria [67]. The other 3 proteins in VacB vaccine: the hypothetical uncharacterized protein ASA_4042, the conjugal transfer mating pair stabilization protein (TraN) ASA_P5G035 and the tolA protein ASA_0744 have not been previously implicated in AS virulence and were chosen for this study on the bases of conservation, functionality and cellular location. Furthermore, the tolA protein in *E*. *coli* has shown to be a major virulence factor [68]. The fish in VacB vaccine group experienced a mortality of 20% suggesting the protective nature (at least some) of the VacB proteins. In addition, the antibody reactivity against ASA_2532 and ASA_P5G035 before the challenge was positively correlated to the survival (Fig 5). Unexpectedly, the fish immunized with VacA vaccine developed significantly elevated antibody levels against few proteins in VacB vaccine—ASA_1342, ASA_P5G035 (after immunization) and ASA_0744 (after challenge) suggesting some unspecific binding of antibodies.

The VacA subunit vaccine included two secreted enzymes: the alpha-amylase (AmyA) ASA_3455 and the endochitinase (chiB) ASA_3320. Both have previously been mentioned as potential virulence factors of AS by Reith [46]. In addition, ASA_3320 has been found abundantly present in supernatant of virulent AS [62, 64]. Another previously mentioned putative virulence factor included in the vaccine was ASA_0826—the RTX protein (asx) that belongs to the RTX family associated with virulence in numerous Gram-negative bacteria [58, 69]. ASA_0826 is an orthologue to a putative virulence factor AHA_3491 in *A*. *hydrophila* [46, 60] and have been found abundantly present in AS supernatant [62–64]. ASA_3883 –a TonB dependent ferric siderophore receptor—is an outer membrane protein and was chosen as vaccine candidate based on its conservation and functionality. In addition, siderophore receptors have been used as potential vaccine candidates against *E*. *coli* in mammalian studies [70–72]. ASA_2321 –a cytochrome C-552 (nrfA) was selected due to functionality and conservation among sub-strains. VacA vaccine proteins were not included in the ELISA analysis due to the lower protective effect and the data on antibody response is not available.

Fish vaccinated with the commercial vaccine was expected to develop a high antibody response against both sonicated bacteria and individual proteins as they were injected with whole cell bacterin, exposing them to a high concentration of a wide array of antigens compared to the fish immunized only with 100–125 μg of potentially immunogenic proteins. The latter fish exhibited a lower antibody response compared to the fish vaccinated with the commercial vaccine but still had high survival. This could indicate that the antibody response is not the only protective factor and that the cellular immunity could be involved in protection. The role of these antigens in vaccine protection should be further elucidated in future studies where proteins are evaluated individually in immunization assays.

Some of the candidate proteins have been previously mentioned as potential virulence factors and protective antigens [16, 46, 62, 64, 65] but the *in vivo* protective potential of those antigens has not been assessed before. This study showed that immunization with previously proposed and few new candidate proteins increased the survival of fish and the survival can be related to the antibody producing protective properties that these proteins may have.

### Future perspectives

Recombinant AS 449 proteins, when applied in experimental vaccines, induce protection against furunculosis and could therefore be considered as vaccine candidates. However the protective input of each antigen needs to be evaluated by single protein immunization to confirm their individual contribution to protection. The relation between antigen concentration and protection should be determined and suitable adjuvants must be selected.

The side-effects in fish immunized with the subunit vaccines adjuvanted with a mixture of Al(OH)_3_ and FIA were not assessed in this study. However, side-effects studies are crucial when testing new vaccine formulations. It is therefore necessary to develop and evaluate the side-effects of suitable adjuvants in future studies.
